# Clinical and biochemical characteristics of 12 Chinese primary hypertrophic osteoarthropathy patients with HPGD mutations

**DOI:** 10.7150/ijbs.71261

**Published:** 2022-06-06

**Authors:** Qi Lu, Yang Xu, Shanshan Li, Zeng Zhang, Jiagen Sheng, Zhenlin Zhang

**Affiliations:** 1 Shanghai Clinical Research Center of Bone Disease, Department of Osteoporosis and Bone Diseases, Shanghai Jiao Tong University Affiliated Sixth People's Hospital, Shanghai, China.; 2 Department of Orthopedic Surgery, Shanghai Jiao Tong University Affiliated Sixth People's Hospital, Shanghai, China.

**Keywords:** Primary hypertrophic osteoarthropathy autosomal recessive 1, HPGD gene, Clinical manifestation, PGE2

## Abstract

Primary hypertrophic osteoarthropathy (PHO) is a rare genetic disease mainly affecting the skeletal and skin. Two genes involved in prostaglandin degradation are known to be responsible for PHO: HPGD and SLCO2A1. HPGD gene mutation can cause PHO autosomal recessive 1 (PHOAR1). The purpose of the present study is to analyze the clinical and biochemical characteristics and HPGD gene mutations of 12 Chinese PHOAR1 patients. Twelve PHOAR1 patients from eleven families, including eleven males and one female, were enrolled in this study. Digital clubbing and periostosis came out to be the most common features, which always occur in the early childhood. We performed HPGD gene analysis and identified six novel (c.1A>G, c.34G>T, c.317T>A, c.475G>T, c.548C>T and c.421+1G>T) and one known (c.310_311delCT) HPGD mutations. The recurrent mutation c.310_311delCT were found in all eleven patients, suggesting it is a hotspot mutation. PHOAR1 patients are considered to have an autosomal recessive inheritance pattern. Here, in addition to nine compound heterozygous patients and two homozygous patients, we found one heterozygous patient and reviewed two heterozygous patients reported in other studies. In terms of biochemical characteristics, our PHOAR1 patients have elevated urinary prostaglandin E2 (PGE2) levels (P<0.001) and decreased urinary prostaglandin E metabolite (PGE-M) levels (P=0.04) compared with healthy controls. The patients' PGE2/PGE-M (E/M) ratio came out to be lower than normal subjects (P<0.001). This study provides a comprehensive description of the clinical phenotypes of Chinese PHOAR1 patients and expands the genotypic spectrum of the disease.

## Introduction

Primary hypertrophic osteoarthropathy (PHO, OMIM 167100) is a rare inherited disease. In 1868, Friedreich first reported this disease in two brothers 1. Later, Touraine, Solente and Golé designated PHO as the primary form of hypertrophic osteoarthropathy 2. PHO mainly involves the skin and skeletal, and the predominant characteristics include pachydermia, digital clubbing, periostosis and joint swelling, sometimes concomitant with arthralgia, affecting joints such as knees, ankles, and wrists. Additional features include hyperhidrosis, acne, acro-osteolysis, anemia, and gastrointestinal abnormalities. PHO should be distinguished from secondary hypertrophic osteoarthropathy (SHO), which has the same skeletal and skin patterns as the primary form. SHO is often associated with impaired lung function. For example, SHO can appear as a paraneoplastic syndrome in patients with lung cancer, which provides clues to the diagnosis. Besides, cardiovascular or other extrathoracic diseases such as inflammatory bowel disease can also cause SHO 3, 4.

Two genes were found to be associated with PHO: HPGD (OMIM 601688) and SLCO2A1 (OMIM 601460), which encode 15-hydroxyprostaglandin dehydrogenase (15-PGDH) and prostaglandin transporter (PGT, also known as solute carrier organic anion transporter family member 2A1), respectively. Uppal et al identified HPGD gene as the causal gene for PHO autosomal recessive type 1 (PHOAR1, OMIM 259100) in 2008 5. Subsequently, in 2012 and 2021, our team found that SLCO2A1 gene deficiency is responsible for both PHO autosomal recessive type 2 (PHOAR2, OMIM 614441) and PHO autosomal dominant (PHOAD, OMIM 167100) 6, 7. These findings provide great breakthrough to clarify the pathogenesis of PHO. Both genes play roles in the prostaglandin metabolism pathway, suggesting the elevated level of prostaglandin E2 (PGE2) may be the pathogenesis. HPGD gene is ubiquitously present in mammalian tissues 8. It localizes on the chromosome 4q34.1 and consists of seven exons. 15-PGDH, the enzyme encoded by HPGD gene, has been considered the key enzyme responsible for the inactivation of prostaglandins. It catalyzes the oxidation of the 15-hydroxyl group of prostaglandins to produce a 15-keto metabolite of greatly reduced biological activities 9. Our previous study demonstrated that both HPGD deficient and SLCO2A1 deficient patients had elevated urinary levels of PGE2 than the normal controls 10. PHO has a male predominance, the skewed male to female ratio is about 9:1. The onset age has a bimodal distribution, peaking during the first year of life in HPGD deficient patients and at puberty in SLCO2A1 deficient patients 11.

In this study, we summarized twelve PHOAR1 patients from eleven families and reviewed all reported patients with HPGD gene mutations. Among them, seven individuals were reported in our previous drug intervention study 12, but the detailed clinical phenotype has not been analyzed before. The other five were additional patients not previously reported. Our present study analyzes their clinical and biochemical characteristics and expands the mutational spectrum of PHOAR1.

## Study subjects and Methods

### Families

**Figure 1:**
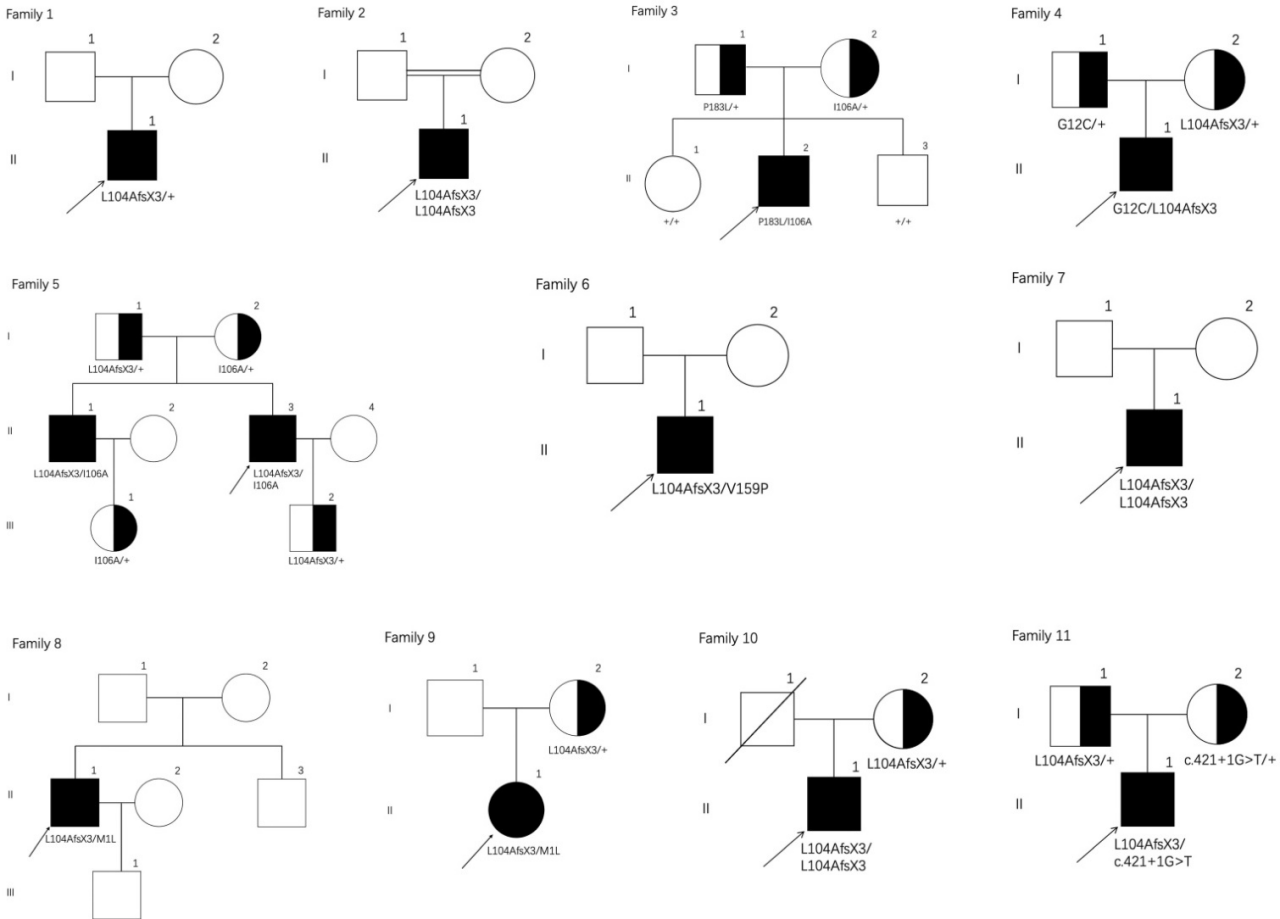
Pedigrees of the eleven families with PHOAR1. Arrows indicate the probands. Filled black circles refer to patients with PHO. Half-black circles refer to healthy relatives with a heterozygous mutation.

Twelve patients from eleven families diagnosed with PHOAR1 according to the standard criteria were involved in this study (Figure 1). Seven individuals were reported before in our previous drug intervention study 12. Here, we especially focused on their clinical features, biochemical characteristics, and mutation analysis. All individuals came from nonconsanguineous families except patient 2. The medical history was collected by retrospective review of medical records and clinical inquiry, complete physical examination and radiological data were obtained and systematically analyzed. Bone mineral densities (BMD) of the lumbar spine and proximal femur were measured with a dual-energy X-ray absorptiometry (Prodigy Advance, GE Lunar Corporation, USA). As described in our previous study 12, the hyponychial angle was used as the index for grading digital clubbing as follows: grade 0 = absent (hyponychial angle <192 degrees), grade 1 = mild (192-202 degrees), grade 2 = moderate (203-207 degrees), and grade 3 = severe (>207 degrees) 13. The scoring system for pachydermia mainly involved the depth of the winkles in the forehead: grade 0 = not furrowed, grade 1 = not furrowed but a ditch between slight swellings can be seen, grade 2 = furrowed but the bottom of the furrow is visible, and grade 3 = deeply furrowed so that the bottom of the furrow is invisible 14.

The study was approved by the Ethics Committee of the Shanghai Jiao Tong University Affiliated Sixth People's Hospital (Ethic Number: 2015-KY-001), and written informed consent was obtained from all the subjects who contributed clinical information and blood and urine samples to the study.

### HPGD gene analysis

For pathogenic gene analysis of affected individuals, genomic DNA was extracted and purified from peripheral blood leukocytes with QuickGene DNA whole blood kit L by Nucleic Acid Isolation System (QuickGene-610L; FUJI FILM, Tokyo, Japan), the exons and exon-intron boundaries of HPGD (NM_000860.5) were amplified through PCR. Direct sequencing was performed using the BigDye Terminator cycle sequencing ready reaction kit, version 3.1 (Applied Biosystems; Foster City, CA, USA), and the reaction was performed using an ABI Prism 3130 automated sequencer (Applied Biosystems). UniProt (http://beta.uniprot.org/), Poly Phen-2 (Polymorphism Phenotyping V2; http://genetics.bwh.harvard.edu/pph2/) and SIFT (Sorting Intolerant from Tolerant; http://sift.jcvi.org/) were used to predict the conservation and pathogenicity of missense mutations found in our patients.

### Biochemical Measurements

Serum bone metabolism markers and sex hormones, including total 25-hydroxyvitamin D (25OHD), intact parathyroid hormone (PTH), Beta-CrossLaps of type 1 collagen containing cross-linked C-telopeptide (β-CTX), osteocalcin (OC), total testosterone, estradiol, luteinizing hormone (LH), follicle stimulating hormone (FSH), and sex hormone-binding globulin (SHBG) were measured using an automated Roche electrochemiluminescence system (Roche Diagnostic GmbH), following both the manufacturer's protocol and specialized assay laboratory quality control procedures. Early morning urine samples were collected at the patients' first visit. Urinary levels of PGE2 and PGE-M were detected using competitive enzyme-linked immunosorbent assays (ELISA; Cayman Chemical, Ann Arbor, MI, USA), which was described in our previous study 12. The values were normalized to creatinine according to the manufacturer's instructions (Item 500141 for PGE2, Item 514531 for PGE-M, Item 500701 for creatinine; Cayman Chemicals, Ann Arbor, MI, USA).

### Statistical analysis

Normally distributed data were expressed as the mean ± standard deviation (SD) while non-normally distributed data were expressed as the median (25th and 75th percentiles). Independent-samples t test and Mann-Whitney U test were used to compare two groups. Pearson correlation test was used to explore the relationships between continuous variables. Statistical significance was set at p <0.05, and SPSS version 23 for Mac (IBM Corp, Armonk, NY, USA) was used for analysis.

## Results

### Clinical manifestations

**Figure 2:**
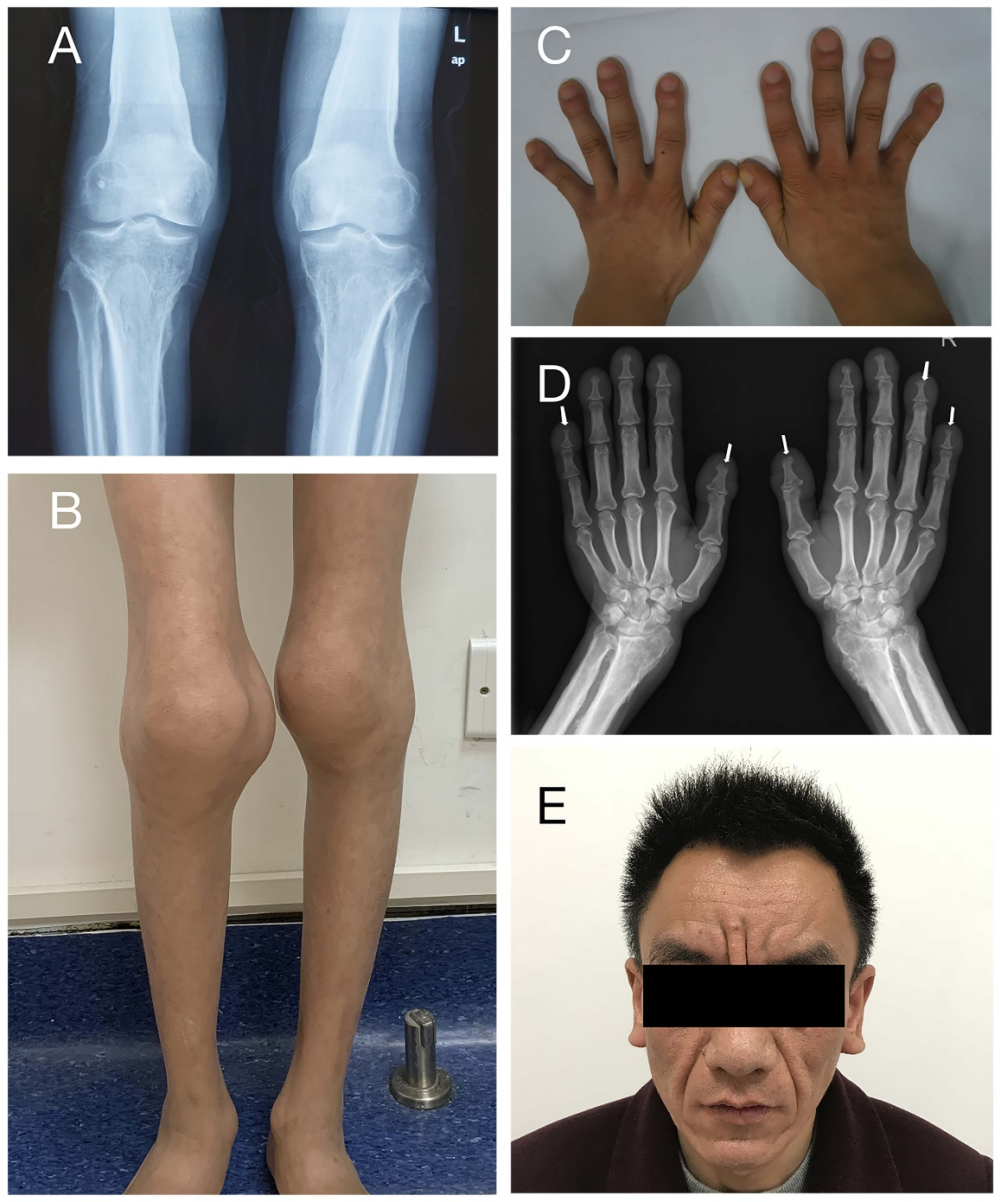
Clinical features of PHOAR1 patients. (**A**) Periostosis in knee joints (Patient 3). (**B**) Joint swelling (Patient 11). (**C**) Digital clubbing (Patient 3). (**D**) Acro-osteolysis (Patient 9). (**E**) Severe facial pachydermia (Patient 9).

A total of 12 patients from 11 families were enrolled in this study, including eleven males and one female, with a median onset age of 2.0 years (range: 1.0-18.0 years). Table 1 summarizes the patients' clinical characteristics. The initial symptom of all patients is digital clubbing, which always occurs in infancy or early childhood. Digital clubbing and periostosis are the most frequent characteristics as they were presented in all patients. The degrees of digital clubbing are listed in Table 1, and more than half patients presented with severe digital clubbing. As for skin manifestations, eight patients have pachydermia and most of them were with a mild degree. Only patient 9 characterizes with severe pachydermia (Figure 2E). Ten patients (83.3%) had joint swelling and four of them (33.3%) were accompanied by arthralgia. The knee joint is most often involved, as all ten individuals complained about knee joint swelling, followed by ankles (seven patients) and wrist (one patient). Patient 10 and patient 12 were found to have patent ductus arteriosus (PDA) at birth. It closed spontaneously in patient 10 at the age of four without any treatment. Patient 12 took surgery and recovered. The incidences of other clinical features are listed in Table 1. None of our patients presented with hypoalbuminemia or gastrointestinal hemorrhage.

### Serum biochemical characteristics and urinary PGE2 and PGE-M levels

**Figure 3:**
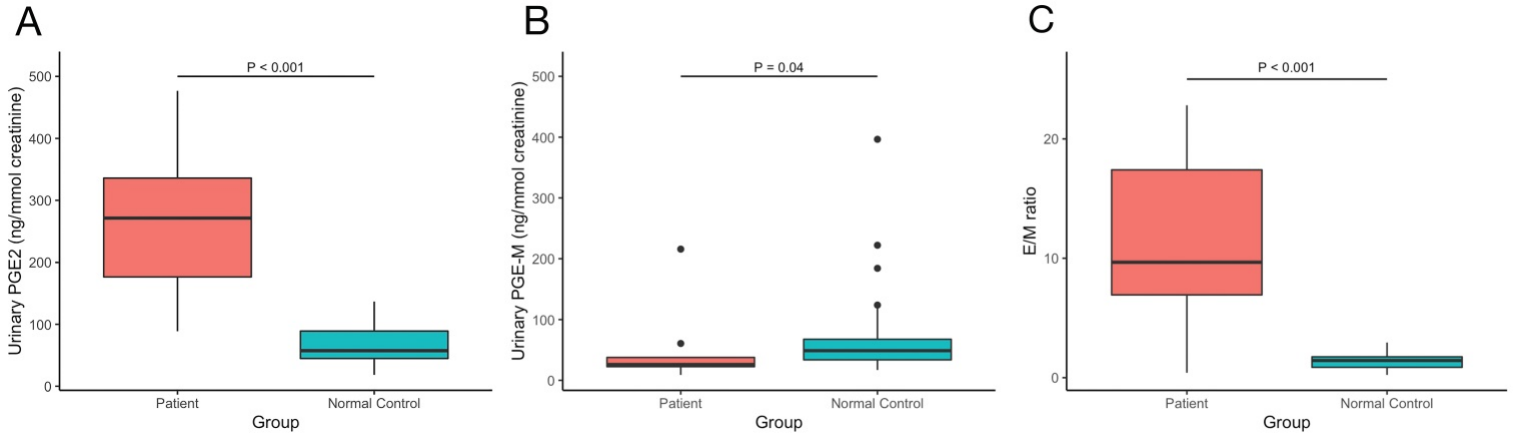
The comparison of urinary (**A**) PGE2, (**B**) PGE-M levels and (**C**) E/M ratio between our PHOAR1 patients and normal controls.

The values of serum bone metabolism markers including β-CTX, OC, PTH, ALP, 25OHD and serum sex hormones including testosterone, estradiol, LH, FSH, SHBG were measured, BMDs at lumber spine, femoral neck and total hip were detected. The results are shown in Table 2. We also measured our patients' urinary levels of PGE2 and PGE-M and calculated the PGE2/PGE-M (E/M) ratio. The results are shown in Figure 3. Compared with normal controls, our PHOAR1 patients had elevated PGE2 levels (271.28 ng/mmol creatinine vs. 57.41 ng/mmol creatinine, p<0.001) and decreased PGE-M levels (26.05 ng/mmol creatinine vs. 48.83 ng/mmol creatinine, p=0.04). And the patients' E/M ratios were higher than healthy controls (9.68 vs 1.44, p<0.001). We did not find any relationships between urinary PGE2 and serum estradiol, FSH, or LH, but the urinary PGE2 level is related to serum testosterone using Pearson correlation analysis (p=0.024).

### HPGD mutations

**Figure 4:**
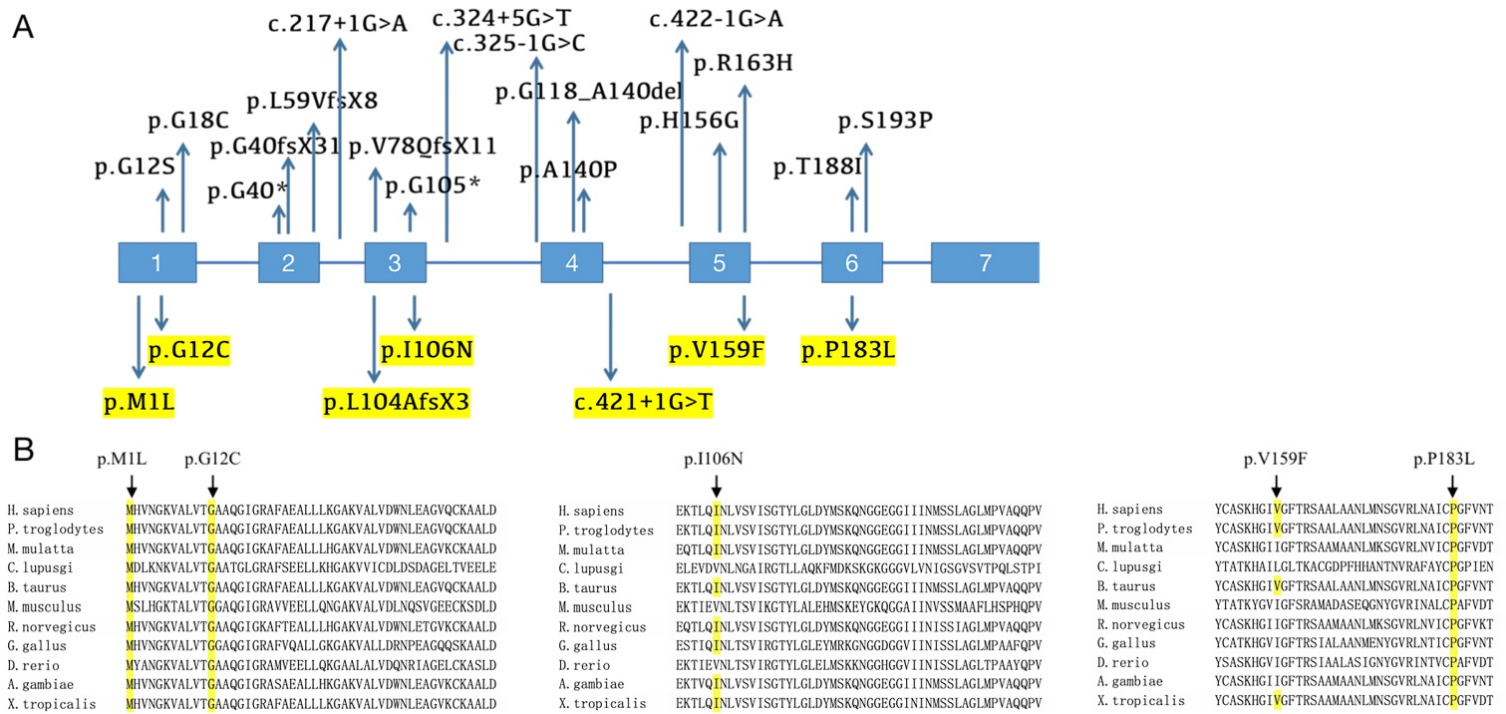
Mutational analysis of PHOAR1 patients. (**A**) Distribution of all mutations in HPGD gene identified so far (the mutation sites identified in our study are highlighted). (**B**) Evolutionary conservation analyses of missense mutations in HPGD gene, as shown by comparing the corresponding sequences of 11 vertebrates.

We screened for HPGD mutations in affected individuals and identified one known and six novel mutations, including five missense mutations, one frameshift mutation and one splicing site mutation. Five mutations were mentioned before in our previous study 12. The mutation sites of each patient are listed in Table 1, and the locations of these mutations on the HPGD gene are shown in Figure 4A (highlight). Eleven patients have the c.310_311delCT mutation in HPGD gene, suggesting it is a hotspot mutation. Among them, one was heterozygous (patient 1), two were homozygous and the remaining eight were compound heterozygous. The only patient without c.310_311delCT mutation had compound heterozygous mutations (c.317T>A/c.548C>T). We also screened for SLCO2A1 mutation and did not identify any pathogenic variants in all the individuals. We verified the mutations in the patients' family members if they agreed (Family 3, 4, 5, 9, 10 and 11). The parents of our probands available for blood samples were carriers carrying monoallelic mutation site of the HPGD gene, and the results are presented in Figure 1. They don't have any PHO symptoms according to the available data. Evolutionary conservation analyses of missense mutations in HPGD gene by comparing the corresponding sequences of different species is shown in Figure 4B. Three amino changes (M1L, G12C and P183L) in HPGD gene were highly conserved according to UniProt. All the point mutations were predicted to be pathogenic by Polyphen-2 or SIFT.

## Discussion

In the present study, the clinical features and laboratory findings of twelve genetically diagnosed Chinese PHOAR1 patients from eleven families were carefully collected and analyzed. We identified six novel (c.1A>G, c.34G>T, c.317T>A, c.475G>T, c.548C>T and c.421+1G>T) and one known (c.310_311delCT) HPGD mutations and described their clinical phenotypes. Although seven individuals were mentioned in our previous drug intervention study 12, our present study provides more detailed information and summarized the features of all twelve PHO patients with HPGD mutations we have up to now. We reviewed all PHOAR1 cases reported so far. A total of 77 cases have been reported to date since HPGD mutations in PHO patients were first identified by Uppal et al in 2008 5, 15-29. We summarized the clinical manifestations of all PHOAR1 patients reported in Table 3. The most common clinical features are digital clubbing and periostosis, which is consistent with our present study. According to our previous study and clinical observation, both PHOAR1 and PHOAR2 patients were affected with periostosis, and the severity did not appear to be strongly related to subtypes. Multilayer or irregular new bone formation occurs mainly at the diaphysis in both subtypes 12. Arthralgia and arthritis are constantly found in PHOAR1 patients, resulting in limitation of motion and decreases of life quality. Knees are most often involved, followed by ankles and wrists. Joint manifestations always occur after digital clubbing and get worse progressively. Pachydermia, one of the triad of PHO, were observed in 52% of the reported PHOAR1 cases, which is much lower than those in PHO patients with SLCO2A1 mutations. More than half (58%) of the patients available for an X-ray examination have acro-osteolysis. Anemia in PHO patients is commonly considered to be due to myelofibrosis or gastrointestinal hemorrhage. However, our two patients presented with anemia don't have gastrointestinal hemorrhage or myelofibrosis. It is well known that PGE2 has the proliferative capacity, enhanced PGE2 synthesis results in increased number of hematopoietic stem cell (HSC), and blocking PGE2 synthesis decreases stem cell numbers, suggesting that PGE2 plays an important role in HSC formation 30. A previous study demonstrated that inhibition of 15-PGDH increases bone marrow PGE2 levels, expands hematopoietic stem cell and progenitor cell numbers, and accelerates hematologic reconstitution after bone marrow transplantation in mice 31.

These studies suggest that prostaglandins play a positive role in hematopoiesis, which is opposite to anemia. Further investigations will be required to answer why PHO patients with elevated PGE2 levels suffer from anemia. Delayed cranial suture closure can presented in affected infants in some of the previously reported cases, this phenotype cannot be seen in our patients since most of them were adults.

The pathogenic HPGD mutations reported so far are listed in Table 3 and presented in Figure 4A. Twenty-five distinct HPGD pathogenic alterations have been identified up to now. The most common mutation appears to be c.310_311delCT, the 2-bp deletion contributes to a frameshift after codon 104 and truncates the protein (p.L104AfsX2), and the lost part includes the proton acceptor site and the putative substrate binding sites 26. This mutation was first reported by Tuysuz et al in a Turkish family 17. Subsequently, 19 additional PHOAR1 patients with c.310_311delCT mutation were reported by four independent studies, including two Turkish patients and seventeen Chinese patients 16, 22, 26, 28. Eleven patients in our present study also have this recurrent mutation, suggesting it is a hotspot mutation, especially in Chinese population. The variant c.175_176del is also a common recurrent mutation. It was reported in eight families and most of them have European origins 5, 27, 29. All the mutation sites reported up to now were summarized in Figure 4A, including eight missense mutations (c.1A>T, c.34G>A, c.52G>T, c.418G>C, c.468T>A, c.488G>A, c.563C>T and c.577T>C), two nonsense mutations (c.118G>T and c.313C>T), four frameshift mutations (c.120delA, c.175_176delCT, c.232_241delinsCA and c.310_311delCT), four splicing site mutation (c.217+1G>A, c.324+5G>A, c.325-1G>C and c.422-1G>A), and one large fragment deletion (deletion of exon 4). Here we identified six novel mutations, including five missense mutations (c.1A>G, c.34G>T, c.317T>A, c.475G>T and c.548C>T), which cause protein changes M1L (identical to the mutation site c.1A>T), G12C, I106N, V159F and P183L, and one splicing site mutation (c.421+1G>T). Most patients are compound heterozygous or homozygous, which fit an autosomal recessive heritance pattern. But in some cases, individuals with monoallelic mutations of HPGD gene can also presented with PHO symptoms, which will be discuss later in this article.

The identification of the causative genes brought us a novel classification of PHO. It can be classified into three subtypes so far: PHOAR1 5, PHOAR2 6 and PHOAD 7. It is important to distinguish between HPGD deficient patients and SLCO2A1 deficient patients in clinical practice as they differ in onset age, sex ratio, clinical features, and biochemical manifestations. The onset age is peaking during the early childhood in HPGD deficient patients and at puberty in SLCO2A1 deficient patients. Although the urinary PGE2 levels were elevated in all three subtypes, it was notably higher in PHOAR2 patients than those in PHOAR1 and PHOAD patients. The urinary levels of PGE-M can also help us distinct PHO subtypes as it usually decreases in HPGD deficient patients and increases in SLCO2A1 deficient patients. And the E/M ratio in HPGD deficient patients is higher than normal people, but in SLCO2A1 deficient patients, it is similar to normal people. Moreover, the clinical manifestation profile differs between subtypes. Pachydermia and clubbing fingers are usually milder in PHOAR1 and PHOAD patients than in PHOAR2 patients. Besides, patients with SLCO2A1 deficiency are more likely to develop gastrointestinal symptoms than those with HPGD deficiency 7, 12.

The pathogenesis of PHO has been well studied since the pathogenic genes were identified. PGE2 degradation requires two steps: first, the prostaglandin transporter mediates cellular PGE2 uptake, and then cytoplasmic oxidation is performed by 15-PGDH 32. Defects of either gene contribute to the elevation of circulation or local levels of prostaglandins (mainly PGE2). We tested urinary PGE2 and PGE-M levels of our patients and found that the defected HPGD gene resulted in elevated urinary PGE2 and decreased urinary PGE-M levels. Skeletal features such as periostosis or acro-osteolysis might be the consequence of elevated PGE2 levels in the circulation or microenvironment since prostaglandins can stimulate both bone resorption and formation 33.

There are gender differences among different subtypes of PHO. It is commonly considered that there is an equal sex ratio in PHOAR1 patients while almost all the PHOAR2 and PHOAD patients are males. The total sex ratio (Female:Male) in reported PHOAR1 cases up to now is 31:46. However, in our cohort, there is only one female patient, which is accordance to two other studies with a Chinese population 16, 28. This makes the sex ratio 3:26 in Chinese population. One explanation might be an incomplete penetrance in females. The skewed sex ratio in PHO patients has long been discovered and discussed. It is proposed that sex hormone may play a potential role, but in PHOAR2 patients, no significant associations of urinary PGE2 or PGE-M with sex hormones, including estradiol, FSH, LH, testosterone, and free testosterone were detected 12. In the present study, we found there is a correlation between levels of urinary PGE2 and serum testosterone, but not other sex hormones. However, this result does not make much sense since the sample size is too small. The relationship between sex hormones and PGE2 metabolism remains to be investigated and data from more patients will be needed. Patient 10 in this study is the only female. She came to our department complaining about arthralgia of her ankles. The patient has special features of cerebral palsy. She was born prematurely, and asphyxia occurred after birth. At one year old, the patient presented with digital clubbing and patent ductus arteriosus was found. The ductus arteriosus closed spontaneously at the age of four. The pain in ankle joints occurred two years before her first visit, and it was obvious several days before menarche and relieved after menarche, which may be explained by the change of PGE2 level related to menstrual cycle. She had obvious periostosis and clubbing fingers, but her skin symptoms were mild. We cannot compare female patients to male patients because of the limited female PHOAR1 case. More PHOAR1 families with both male and female patients are required to help us reveal the effect of gender on PHOAR1, which will be our future direction.

Both dominant and recessive inheritance patterns have been suggested in PHO patients. Recently, our team identified a novel dominant pattern of PHO with SLCO2A1 mutation 7. Up to now, all patients with HPGD mutations showed an autosome recessive inheritance pattern except three Chinese individuals with c.310_311delCT mutation reported in Yuan's study and ours 16, indicating an autosomal dominant inheritance pattern may also exists in PHO patients with HPGD mutations. The heterozygous patient in our study had mild symptoms which is listed in Table 1 (patient 1). We detected his urinary levels of PGE2 and PGE-M, which were 88.77 ng/mmol creatinine and 215.78 ng/mmol creatinine, respectively. His urinary PGE2 level was not elevated. And the PGE-M level was higher than normal controls, which confused us. Maybe there exist other enzymes that can degrade prostaglandins. Unfortunately, we do not have any information about his parents and more families or cases with monoallelic HPGD mutation will be needed to clarify this phenomenon.

Since the elevated PGE2 level is considered to be the pathogenesis, the selective cyclooxygenase-2 (COX-2) inhibitor which can suppress PGE2 biosynthesis was used for treatment. Two clinical trials confirmed the efficacy and safety of etoricoxib 12, 34. Seven PHOAR1 patients were recruited in our previous trial 12. The results showed that the urinary PGE2 levels in our patients were decreased after 6-month etoricoxib treatment and the biochemical bone markers have also been improved. The PHO symptoms including pachydermia and digital clubbing were relieved at the evaluation at 6 months. However, periostosis did not improve at 6 months according to the X-ray examination.

Recently, Palla et al pointed out that 15-PGDH may influence the muscle strength and identified elevated 15-PGDH as a hallmark of aged muscles 35. The inhibition of 15-PGDH increases aged muscle mass, strength, and exercise performance in mice, indicating that it may be a potential treatment of sarcopenia. We wonder if this phenotype can also be presented in our patients with 15-PGDH deficiency. We contacted our patients and asked if they think their muscle strength is superior to that of their peers, but they all denied that. On the contrary, some of these patients have slender limbs and look weak. We measured their BMIs, with a median of 21.26 kg/m^2^, which is in the normal range. However, the exact muscle strength data were unavailable because we did not pay much attention to their muscle strength before. We plan to collect more detailed data and use precise methods to measure their muscle strength for further investigation.

In conclusion, here we described eleven PHOAR1 families. The early onset digital clubbing and periostosis are shown to be the most frequent symptoms. Mutational analysis was performed and seven mutations in HPGD gene were identified. Among them, six were novel mutations. One recurrent mutation c.310_311delCT were found in eleven patients, indicating it is a hotspot mutation in Chinese population. Elevated urinary PGE2 and decreased urinary PGE-M levels were found in our patients. Etoricoxib, a selected COX-2 inhibitor, was used for treatment and was beneficial to our patients. This study analyzed clinical and biochemical characteristics of PHOAR1 patients and expands the mutational spectrum.

## Figures and Tables

**Table 1:** Clinical and genetic features of PHOAR1 patients

Family	F1	F2	F3	F4	F5		F6	F7	F8	F9	F10	F11	Total
Patient	P1	P2	P3	P4	P5	P6	P7	P8	P9	P10	P11	P12	
Sex	male	male	male	male	male	male	male	male	male	female	male	male	11/1*
Age	42	36	33	35	53	58	32	39	48	31	29	12	35.5 (12.0-58.0)**
Onset age	15	1	16	2	18	12	1	2	4	1	1	1	2.0 (1.0-18.0)**
Consanguineous	-	+	-	-	-	-	-	-	-	-	-	-	1/12 (8.3%)
Clubbing Fingers	mild	severe	moderate	severe	severe	mild	severe	+	severe	moderate	severe	severe	12/12 (100%)
Pachydermia	mild	mild	-	mild	mild	-	mild	+	severe	-	mild	-	8/12 (66.7%)
Periostosis	+	+	+	+	+	+	+	+	+	+	+	+	12/12 (100%)
Joint swelling	-	+	+	+	+	+	+	-	+	+	+	+	10/12 (83.3%)
Arthralgia	-	-	-	+	+	-	-	-	-	+	+	-	4/12 (33.3%)
Acro-osteolysis	-	-	-	+	+	-	+	-	+	-	-	-	4/12 (33.3%)
Hyperhidrosis	-	-	-	+	+	-	+	+	+	+	-	+	7/12 (58.3%)
Acne	-	-	-	+	+	-	+	-	-	-	+	-	4/12 (33.3%)
Seborrhea	+	-	+	+	+	-	+	+	+	+	+	-	9/12 (75%)
Anemia	-	-	+	-	-	-	-	-	-	-	-	+	2/12 (16.7%)
Patent ductus arteriosus	-	-	-	-	-	-	-	-	-	+	-	+	2/12 (16.7%)
Watery diarrhea	-	-	+	+	+	+	-	-	-	-	-	-	4/12 (33.3%)
DNA change	c.310_311delCT(heterozygous)	c.310_311delCT/c.310_311delCT	c.317T>A/c.548C>T	c.310_311delCT/c.34G>T	c.310_311delCT/c.317T>A	c.310_311delCT/c.317T>A	c.310_311delCT/c.475G>T	c.310_311delCT/c.310_311delCT	c.310_311delCT/c.1A>G	c.310_311delCT/c.1A>G	c.310_311delCT/c.310_311delCT	c.310_311delCT/c.421+1G>T	/
Parents	NA	NA	F: c.548C>T M: c.317T>A	F: c.34G>T M: c.310_311delCT	F: c.310_311delC M: c.317T>A		NA	NA	NA	F:NA M: c.310_311delCT	F:NA M: c.310_311delCT	F: c.310_311delC M: c.421+1G>T	/

NA, not available; F, father; M, mother. *male/female; **median (range).

**Table 2:** Biochemical features of PHOAR1 patients

Patient	P1	P2	P3	P4	P5	P6	P7	P8	P9	P10	P11	P12	median (25^th^ and 75^th^ percentiles) or mean ± SD
Height (cm)	NA	NA	167.00	173.90	181.10	172.90	174.50	158.00	173.20	153.40	172.50	137.00	172.70 (156.85, 174.05)^a^
Weigh (kg)	NA	NA	52.00	69.00	83.00	70.50	57.00	49.00	60.00	53.00	80.00	23.50	59.70 ± 17.30^b^
BMI (kg/m^2^)	NA	NA	18.65	22.82	25.31	23.58	18.72	19.63	20.00	22.52	26.89	12.52	21.06 ± 4.10
PGE2 (ng/mmol creatinine)	88.77	NA	185.64	261.50	370.65	281.06	476.64	NA	324.38	NA	149.53	NA	271.28 (158.56, 359.08)
PGE-M (ng/mmol creatinine)	215.78	NA	25.68	30.02	60.67	26.41	25.18	NA	14.22	NA	8.85	NA	26.05 (16.96, 53.00)
E/M ratio	0.41	NA	7.22	8.71	6.11	10.64	18.93	NA	22.81	NA	16.90	NA	9.68 (6.39, 18.42)
ALP (U/L)	NA	NA	68.00	86.00	113.00	88.00	78.00	82.00	84.00	61.00	102.00	189.00	85.00 (75.50, 104.75)
β-CTX (ng/L)	NA	NA	NA	NA	132.00	310.00	718.00	726.20	412.10	NA	861.20	2033.00	741.79 ± 625.74
OC (ng/mL)	NA	NA	NA	NA	11.95	27.58	23.78	35.26	24.16	NA	37.24	146.90	27.58 (23.78, 37.24)
PTH (pg/mL)	NA	NA	41.47	NA	42.43	50.67	34.31	39.14	49.41	35.66	57.05	21.15	41.25 ± 10.57
25OHD (ng/mL)	NA	NA	16.74	NA	19.60	14.78	26.42	28.99	18.30	22.29	14.29	22.89	20.48 ± 5.09
Estradiol (pmol/L)	NA	NA	95.00	NA	146.72	144.06	276.06	155.75	220.5	474.16	140.78	NA	151.24 (143.24, 234.39)
FSH (IU/L)	NA	NA	4.68	NA	8.47	5.25	1.12	9.09	4.42	4.08	3.78	1.02	4.65 ± 2.77
LH (IU/L)	NA	NA	2.86	NA	6.40	3.60	3.21	3.71	4.75	4.74	6.26	0.28	3.98 ± 1.87
Testosterone (nmol/L)	NA	NA	4.64	NA	15.94	7.63	18.22	12.23	8.59	1.48	8.09	0.12	8.55 ± 6.11
SHBG (nmol/L)	NA	NA	NA	NA	45.50	34.50	35.00	58.40	67.00	42.60	20.10	119.80	52.86 ± 30.71
L1-L4 BMD (g/cm^2^) (Z score)	NA	NA	1.133 (0.8)	1.273 (0.5)	1.140 (0.4)	NA	1.199 (0.5)	1.173 (0.9)	1.340 (2.4)	1.374 (2.2)	1.089 (-0.4)	0.601 (0.1^c^)	1.147 ± 0.226 (0.8 ± 0.9)
Femoral neck BMD (g/cm^2^) (Z score)	NA	NA	0.906 (-0.3)	0.964 (0.0)	0.920 (-0.2)	NA	1.014 (0.5)	1.047 (0.7)	0.967 (0.3)	1.250 (2.7)	0.745 (-2.1)	0.615 (-0.1^c^)	0.936 ± 0.180 (0.2 ± 1.2)
Total hip BMD (g/cm^2^) (Z score)	NA	NA	0.997 (0.2)	1.219 (2.0)	1.054 (0.5)	NA	1.040 (0.8)	1.031 (0.3)	1.058 (0.6)	1.333 (2.8)	0.758 (-1.9)	0.649 (-0.1^c^)	1.015 ± 0.208 (0.6 ± 1.3)

NA, not available. a. Non-normally distributed data are shown as the median (25^th^ and 75^th^ percentiles). b. Normally distributed data are shown as the mean ± SD. c. The Z score at L1-L4, femoral neck and total hip of young patients were calculated by comparison with the age-specific BMD reference value of Chinese children.'

**Table 3:** Summary of phenotypes and genotypes in reported patients with HPGD mutations

Reference	Uppal et al.	Seifert et al	Tariq et al	Yuksel-Konuk et al	Diggle et al	Sinibaldi et al	Bergmann et al	Tuysuz et al	Erken et al	Nakazawa et al	Yuan et al	Chen et al	Khan et al	Stephan et al	Pang et al	Radhakrishnan et al	Total	The present study
Sex ratio (F/M)	8/5	3/1	7/4	5/1	3/5	0/1	0/3	0/2	1/1	0/1	1/8	1/6	2/2	0/1	0/1	0/4	31/46	1/11
heterozygous	0/13	0/4	0/11	0/6	0/8	0/1	0/3	0/2	0/2	0/1	2/9	0/7	0/4	0/1	0/1	0/4	2/77 (2.6%)	1/12 (8.3%)
Clubbing finger	13/13	4/4	11/11	6/6	8/8	1/1	3/3	2/2	2/2	1/1	9/9	7/7	4/4	1/1	1/1	4/4	77/77 (100%)	12/12 (100%)
Periostosis	8/8	0/2	0/2	4/4	4/4	1/1	3/3	2/2	2/2	1/1	8/9	0/7	1/1	NE	1/1	3/4	38/51 (74.5%)	12/12 (100%)
Pachydermia	9/13	0/4	0/11	4/6	1/8	0/1	2/3	0/2	2/2	1/1	8/9	7/7	2/4	1/1	1/1	1/2	39/75 (52%)	8/12 (66.7%)
Joint swelling	5/13	0/4	0/11	3/6	5/8	0/1	1/3	2/2	2/2	0/1	7/9	1/7	4/4	0/1	1/1	2/4	33/77 (42.9%)	10/12 (83.3%)
Arthralgia	10/13	1/4	0/11	NM	3/8	1/1	1/3	2/2	2/2	1/1	0/9	0/7	2/4	0/1	1/1	4/4	28/71 (39.4%)	4/12 (33.3%)
Acro-osteolysis	8/8	2/2	0/2	4/4	2/4	1/1	2/3	0/2	2/2	NE	1/9	1/7	1/1	NE	1/1	4/4	29/50 (58%)	4/12 (33.3%)
Hyperhidrosis	12/13	4/4	0/11	5/6	6/8	1/1	2/3	2/2	2/2	1/1	7/9	NM	4/4	1/1	1/1	0/2	48/68 (70.6%)	7/12 (58.3%)
Patent ductus arteriosus	4/13	1/4	0/11	1/6	2/8	0/1	0/3	1/2	0/2	0/1	0/9	2/7	0/4	0/1	0/1	1/4	12/77 (15.6%)	2/12 (16.7%)
Anemia	NM	NM	0/11	0/6	NM	1/1	NM	0/2	1/2	0/1	0/9	0/7	0/4	0/1	0/1	NM	2/45 (4.4%)	2/12 (16.7%)
Acne	4/13	0/4	0/11	NM	NM	1/1	2/3	0/2	2/2	NM	NM	NM	2/4	0/1	0/1	NM	11/42 (26.2%)	4/12 (33.3%)
Seborrhea	10/13	0/4	0/11	NM	NM	1/1	2/3	0/2	NM	1/1	1/9	NM	4/4	0/1	1/1	NM	20/50 (40%)	9/12 (75%)
DNA change	c.418G>C* c.232_241delinsCA* c.175_176delCT*	c.52G>T* c.120delA*	c.577T>C*	c.418G>C c.1A>T*	c.120delA c.175_176delCT c.325-1G>C*	c.217+1G>A*	c.118G>T* c.563C>T* c.175_176delCT	c.310_311delCT*	c.310_311delCT	c.422-1G>A*	c.310_311delCT c.488G>A*	c.310_311del CT c.324+5G>A* a Deletion of exon 4*	c.577T>C	c.468T>A*	c.310_311delCT	c.34G>A* c.418G>C c.313C>T*	/	c.310_311delCT c.317T>A* c.548C>T* c.475G>T* c.34G>T* c.1A>G* c.421+1G>T*

NM, not mentioned; NE, not evaluated. *Novel mutations first identified by the referenced study.

## Abbreviations

PHO: primary hypertrophic osteoarthropathy
SHO: secondary hypertrophic osteoarthropathy
PHOAR1: primary hypertrophic osteoarthropathy, autosomal recessive 1
PHOAR2: primary hypertrophic osteoarthropathy, autosomal recessive 2
PHOAD: primary hypertrophic osteoarthropathy, autosomal dominant
PGE2: prostaglandin E2
PGE-M: prostaglandin E metabolite
E/M ratio: PGE2/ PGE-M ratio
SLCO2A1: solute carrier organic anion transporter family member 2A1
PGT: prostaglandin transporter
HPGD or 15-PGDH: 15-hydroxyprostaglandin dehydrogenase
BMD: Bone mineral density
25OHD: 25-hydroxyvitamin D
PTH: parathyroid hormone
β-CTX: Beta-CrossLaps of type 1 collagen containing cross-linked C-telopeptide
OC: osteocalcin
LH: luteinizing hormone
FSH: follicle stimulating hormone
SHBG: sex hormone-binding globulin
COX-2: cyclooxygenase-2
HSC: hematopoietic stem cell
